# Metastatic, Her-2 Amplified Lacrimal Gland Carcinoma with Response to Lapatinib Treatment

**DOI:** 10.1155/2015/262357

**Published:** 2015-02-02

**Authors:** Trevor Dennie

**Affiliations:** Multicare Regional Cancer Center, 121 N. Division Street, No. 100, Auburn, WA 98001, USA

## Abstract

Carcinoma of the lacrimal gland is a rare malignancy, limiting opportunities to develop new therapeutic regimens through clinical trials. There are no standard guidelines on optimal treatment of lacrimal gland carcinoma. In addition, lacrimal gland carcinoma includes several different subtypes with distinct behavior and response to treatment, further complicating treatment. Overexpression of the Her-2/neu protein, a potential target for new therapeutic agents, has previously been described in lacrimal gland carcinoma; however, there are no published reports regarding treatment of lacrimal gland cancer with Her-2 directed medications. This case report describes treatment of a patient with metastatic lacrimal gland carcinoma with lapatinib, an oral agent with activity against Her-2/neu amplified malignancies. In this case, Her-2 overexpression was confirmed by biopsy of a metastatic site. PET imaging obtained 6 months after the initiation of lapatinib showed evidence of a partial response to treatment, although the patient later developed progressive neurologic complications related to her malignancy and ultimately died. Lapatinib and other Her-2 targeted agents may represent an effective therapeutic option for this rare malignancy, in cases of metastatic disease.

## 1. Introduction

Neoplasms of the salivary and lacrimal glands encompass a wide spectrum of disease. More than 30 different subtypes were described in the 2005 WHO Classification of Tumours: Pathology and Genetics of Head and Neck Tumours, though benign tumors (adenomas) and adenoid cystic carcinoma typically account for a large fraction of cases [1]. Adenocarcinoma of the lacrimal gland is a rare malignancy. The estimated incidence of all tumors of the lacrimal gland, including benign lesions, is less than one case per million per year [2]. Adenocarcinoma is estimated to represent 5–10% of malignant cases. There are no standard guidelines for treating lacrimal gland adenocarcinoma; in fact, the National Comprehensive Cancer Network (NCCN) does not publish treatment guidelines for lacrimal gland malignancies or even for ocular tumors as a group. Treatment is often derived from studies on therapy for salivary gland tumors. Malignancies of the eye were not even included in the Tumor, Node, Metastasis (TNM) staging system until the 4th edition. The latest (7th) edition includes staging criteria for 3 sites of periocular carcinoma—the eyelids, conjunctiva, and lacrimal gland—however, these criteria have yet to be validated by clinical studies [3].

## 2. Case Presentation

A 53-year-old woman was seen for transfer of care for metastatic lacrimal gland carcinoma. She had been diagnosed 2 and 1/2 years earlier in another state. She originally presented with progressive headaches, blurred vision, and right eye proptosis. CT imaging showed 2 separate masses involving the right orbit (a 2.5–3 cm mass under right orbital roof in the lacrimal area, also a 1 cm mass in the right orbital apex and superior orbital fissure) with possible intracranial extension. MRI imaging showed a right superior lateral orbital mass apparently originating from the lacrimal gland, invading into surrounding bone and possibly the brain parenchyma. The patient underwent subtotal resection of the locally advanced right orbital mass. The pathologic diagnosis was metastatic carcinoma of lacrimal gland origin. Immunohistochemistry was positive for keratin, CK7, and mammaglobin; negative for CK20, TTF-1, BRST-2 or estrogen or progesterone receptors.

The patient then received adjuvant radiation treatment to the right orbital area. She developed symptoms suggestive of metastatic disease less than a year later. MRI imaging of the thoracic spine showed 3 separate areas of intramedullary metastatic disease, at the T1 level, T6-7, and T12. There was no metastatic disease seen in the lumbar spine by MRI, and CT imaging of the chest and abdomen done at the same time also showed no evidence of metastases. The patient was again treated with radiotherapy. She later received radiation to new metastatic sites in the left cerebellum and an intramedullary lesion in the cervical spine.

PET scan done following transfer of care showed evidence of disease progression in the cervical spine and proximal thoracic spine, as well as bilateral lung nodules and thoracic lymphadenopathy suggestive of systemic metastases. A biopsy was performed of one of the lung nodules, confirming metastatic adenocarcinoma. Due to the intramedullary location of spine mets, as well as prior radiotherapy to these sites, the patient was not considered a candidate for palliative surgery or radiation. Additional testing performed on the lung biopsy specimen showed no evidence of an epidermal growth factor receptor (EGFR) mutation; however, Her-2/neu expression was found to be amplified by FISH assay (Her-2/cen 17 ratio of 6.0). The patient was started on lapatinib, an oral tyrosine kinase inhibitor with systemic and central nervous system (CNS) activity against both Her-2/neu and EGFR, at a dose of 1500 mg daily. She tolerated lapatinib without significant side effects. CT imaging after 2 months of treatment showed stable disease.

After approximately 5 months of treatment, the patient developed progressive weakness in both lower extremities. MRI imaging showed a moderate increase in size of the intramedullary lesion at T1, as well as increased cord edema and a syrinx extending to the C3 level. The findings were felt to be indeterminate for progression of malignancy versus radiation myelitis from prior radiotherapy. PET imaging was ordered to further evaluate this lesion. The PET study showed grossly stable size of multiple metastatic lesions but significant decrease in standardized uptake value (SUV) at all disease sites, consistent with a response to treatment. For example, the SUV of the largest lung lesion had decreased from 28.8 to 6.2, while the SUV of the T1 lesion had decreased from 18.6 to 6.6. Lapatinib was continued, based on the evidence of response and also as the patient was not considered a candidate for more aggressive treatment such as high-dose IV methotrexate. She was also treated with oral dexamethasone. Unfortunately, the patient's lower extremity weakness progressed to the point of paraplegia, and she had a steady clinical decline related to this. She ultimately died approximately 10 months after starting lapatinib and more than 4 years after original diagnosis.

## 3. Discussion

Adenocarcinoma of the lacrimal gland is a rare malignancy. The estimated incidence of all tumors of the lacrimal gland, including benign lesions, is less than one case per million per year [2]. Of the malignant subtypes, the most common is adenoid cystic carcinoma. Adenocarcinoma is estimated to represent 5–10% of malignant cases. Overexpression of the Her-2 protein in lacrimal gland tumors was first reported more than 20 years ago [4]; however, limited additional data has been published since then. In one case series published in 2013, specimens from 5 patients diagnosed with primary ductal adenocarcinoma of the lacrimal gland, an uncommon subtype, were tested for Her-2 overexpression; this was positive in 3 cases [5]. Three of the patients in this series died within a 5-year follow-up period. It is unclear if any of these patients were treated with Her-2 directed agents, or other systemic therapy.

This is the first published case report of a patient with metastatic lacrimal gland adenocarcinoma to be treated with, and respond to, an agent targeted at Her-2 overexpression. In this case, lapatinib was chosen over other agents due to its CNS penetration. Until the diagnosis of lung metastases, the patient's metastatic disease had been limited to the brain and spinal cord, and she had only received radiotherapy to disease sites within the CNS without any systemic treatment. The patient tolerated lapatinib well. Approximately 6 months after starting lapatinib, PET imaging was repeated to better define a progressive intramedullary lesion at the T1 level. This showed evidence of a response to treatment by decreased SUV uptake at multiple sites of metastatic disease, though the metastatic lesions had not decreased in size appreciably. However, the patient had progressive neurologic deficits related to the T1 lesion and associated cord edema and syrinx, ultimately resulting in declining performance status and paraplegia.

In summary, pharmaceutical agents directed at Her-2 overexpression may be a therapy option in patients with metastatic lacrimal gland adenocarcinoma. This is a rare malignancy, limiting the ability to conduct clinical studies, and data regarding treatment options are quite limited. A prior pathologic case series determined that Her-2 overexpression was a common feature of primary ductal adenocarcinoma of the lacrimal gland, a rare subtype of lacrimal gland adenocarcinoma. In this case, further pathologic testing was not performed to determine the subtype of adenocarcinoma, as this did not appear to have any bearing on management or therapeutic options. It is possible that in this case, the patient may have had a longer sustained response if systemic treatment had been initiated earlier, when she was first diagnosed with metastatic disease in the CNS. As it was, she did not begin treatment with lapatinib for more than 2 years from initial diagnosis of metastatic disease. There is a growing list of FDA-approved agents targeted at Her-2 overexpression, including not only lapatinib but trastuzumab (Herceptin), ado-trastuzumab emtansine (Kadcyla), and pertuzumab (Perjeta).
